# A Neuro-oncologist’s Perspective on Management of Brain Metastases in Patients with EGFR Mutant Non-small Cell Lung Cancer

**DOI:** 10.1007/s11864-017-0466-0

**Published:** 2017-04-08

**Authors:** Tresa McGranahan, Seema Nagpal

**Affiliations:** Department of Neurology, Stanford University School of Medicine, 300 Pasteur Drive, Stanford, CA 94305 USA

**Keywords:** Non-small cell lung cancer (NSCLC), Epidermal growth factor receptor (EGFR), Tyrosine kinase inhibitors (TKI), Brain metastasis, Whole brain radiation therapy (WBRT), Radiosurgery (SRS)

## Abstract

Management of non-small cell lung cancer (NSCLC) with brain metastasis (BrM) has been revolutionized by identification of molecular subsets that have targetable oncogenes. Historically, survival for NSCLC with symptomatic BrM was weeks to months. Now, many patients are surviving years with limited data to guide treatment decisions. Tumors with activating mutations in epidermal growth factor receptor (EGFRact+) have a higher incidence of BrM, but a longer overall survival. The high response rate of both systemic and BrM EGFRact+ NSCLC to tyrosine kinase inhibitors (TKIs) has led to the rapid incorporation of new therapies but is outpacing evidence-based decisions for BrM in NSCLC. While whole brain radiation therapy (WBRT) was the foundation of management of BrM, extended survival raises concerns for the subacute and late effects radiotherapy. We favor the use of TKIs and delaying the use of WBRT when able. At inevitable disease progression, we consider alternative dosing schedules to increase CNS penetration (such as pulse dosing of erlotinib) or advance to next generation TKI if available. We utilize local control options of surgery or stereotactic radiosurgery (SRS) for symptomatic accessible lesions based on size and edema. At progression despite available TKIs, we use pemetrexed-based platinum doublet chemotherapy or immunotherapy if the tumor has high expression of PDL-1. We reserve the use of WBRT for patients with more than 10 BrM and progression despite TKI and conventional chemotherapy, if performance status is appropriate.

## Introduction

Metastatic lung cancer remains a devastating diagnosis, due in part to the high incidence of brain involvement; however, advances in targeted therapy are prolonging survival [1••]. Lung cancer with brain metastasis (BrM) remains an incurable disease and the goals of care are optimizing quality of life (QOL) and maximizing duration of disease control. Unfortunately, given the toxicity of many treatments, the presence of neurologic symptoms limited the therapies that could be offered to patients. However, the availability of more tolerable and effective agents is changing the historical approach to BrM.

The treatment of non-small cell lung cancer (NSCLC), which accounts for 85% of lung cancer diagnosis, is being transformed by the identification of oncogenic driver mutations [2•]. Mutations are present in 60% of adenocarcinomas and the current standard of care is to evaluate molecular markers, particularly mutations in epidermal growth factor receptors (EGFR), in advanced disease. There is expanding evidence that these are unique tumors in regard to prognosis, incidence of metastases, and treatment response. While lower incidence, there are other clinically applicable driver mutations that have targeted therapies, including ALK, ROS-1, BRAF, MEF, and RET-1, that will not be addressed in this paper as it is uncommon for patients to express more than one driver mutation [3].

The feedback loop from bench to bedside has perhaps never been as productive as with EGFR in NSCLC. High expression of EGFR has been recognized in multiple malignancies with significant expression in lung cancer with BrM [4, 5]. Small molecules, tyrosine kinase inhibitors (TKIs) were developed to target these receptors. A subpopulation of Asian, female, non-smokers with adenocarcinoma had a striking sustained response rate (RR). Evaluation of EGFR genotype in the highly responsive subpopulation lead to identification of multiple mutations in the activating domain of EGFR (EGFRact+). These mutations have higher affinity for first generation TKIs than wildtype EGFR (EGFRwt) [6, 7]. With increased screening for EGFRact+ in NSCLC, it is clear that the phenotype of Asian, non-smoking women with adenocarcinoma only accounts for about 60% of patients. Overall frequency of EGFRact+ in Europe is 15% and 22% in North America [8•]. Up to 25% patients with EGFRact+ NSCLC are current smokers [9]. NSCLC expressing EGFRact+ are distinct with a higher incidence of BrM, greater overall survival and possibly increased response to radiotherapy [10]. Within the EGFRact+ mutations, retrospective studies have also suggested that specific mutations may be both predictive and prognostic marker for better outcome [11].

Despite changes in the treatment of systemic NSCLC, recommendations for management of BrM are based on mixed populations of solid tumors. While there is limited evidence to support whole brain radiation (WBRT) compared to best supportive care, it has been the foundation of management in patients with multiple BrM for many decades. Given advancements in systemic therapy as well as radiosurgery (SRS), the late cognitive effects associated with WBRT is of increasing significance on QOL [12•]. Additionally, the tolerability of TKIs enables these agents to be offered to most patients regardless of performance status, expanding the populations eligible for subsequent local therapy.

There remain multiple unanswered questions in the management of BrM in EGFRact+ NSCLC. This paper reflects our current practice based on the evidence available. The development of new therapies and ongoing clinical trials examining the combination of local and systemic therapies will continue to transform the management of BrM in EGFRact+ NSCLC in the coming years.

## Systemic therapy

Traditionally, systemic therapy had a limited role in the management of BrM, largely due to limitations in blood-brain barrier (BBB) permeability as well as the risk of toxicity to surrounding healthy brain tissue. Advances in conventional chemotherapy as well as TKIs are changing the management of BrM. TKIs are first line therapy for advanced systemic EGFRact+ NSCLC and given tolerability and rapid action of these agents, we offer these medications to all EGFRact+ NSCLC patients regardless of performance status. We carefully monitor for CNS response and progression with MRIs. In our experience, response to these agents enables subsequent therapies that would not otherwise be tolerated. Platinum doublet chemotherapy utilizing pemetrexed is reserved for disease refractory to TKIs. We typically reserve immunotherapy until progression despite conventional chemotherapy, unless there is high tumor expression of PDL-1. We utilize bevacizumab (BEV) for the management of steroid refractory cerebral edema as well as radiation necrosis; recognizing it may also have an oncolytic effect.

### EGFR tyrosine kinase inhibitors

Consideration of current use of EGFR TKIs requires greater understanding of their brief and transformative history. When initially studied, TKIs had less than 20% RR in an unselected population. Subsequent evaluation of tumor genomics lead to the identification of driver mutations in EGFR (EGFRact+) [6, 7]. Driver mutations are genetic alterations found within a tumor that result in constitutively active forms of signaling proteins and lead to sustained tumorgenesis. The most common EGFR activating mutations are deletions of exon 19 (exon19del) and point mutations of exon 21 (L858R) [13]. On exon 20, T790M is a resistance mutation that is seen in TKI naïve patients and in 60% of patients who develop resistance to TKIs [14]. This has led to a third generation of TKIs specifically targeting T790M. While there is data to support lower rates of CNS progression in EGFRact+ NSCLC treated with TKI compared to conventional chemotherapy, the brain is a frequent site of disease recurrence after an initial response to TKIs [15, 16]. Currently available agents were not designed for BBB penetration; however, there are multiple agents in the pipeline designed for increased CNS penetration.

The use of TKIs in patients with BrM is expanding without randomized trials to guide treatment strategies (Table 1). As with most early clinical trials, patients with active BrM were largely excluded from the initial trials of TKIs. The currently available studies are limited in that they are small and predominately single arm or retrospective studies with variable evaluation for EGFRact+. While CNS concentrations of all currently available agents are lower than plasma, CNS disease in EGFRact+ patients have high RR to TKIs. There are ongoing clinical trials to specifically evaluate the use of these agents in patients with BrM and active CNS disease as well as evaluations of new agents designed specifically for increased BBB penetration.

**Table 1:** Prospective trials of TKIs in brain metastasis

TKI		Type of study	Prior therapy	Concurrent therapy	EGFR status	*n*	OS
Afatinib	[41]	Post hoc phase 3	37% prior XRT		All EGFRact+	91	19.78 m
Erlotinib	[32]	Phase I	21% prior CT + XRT	WBRT	NS	11	4.4 m
Erlotinib	[103]	Phase 2	Prior CT		8/23 EGFRact+	48	18.9 m
Erlotinib	[48]	Phase 2	Prior TKI + XRT		6/7 EGFRact+	7	2.9 m
Erlotinib	[33•]	Phase 2	Prior CT	WBRT	1/35 EGFRact+	80	3.4 m
Erlotinib	[104]	Phase 2	Prior CT or CT + XRT	WBRT	11/23 EGFRact+	23	10.7 m
Erlotinib	[34]	Phase 2, multiI	52% prior CT, 10% prior SRS	WBRT	17/26 EGFRact+	40	19.1 m
Erlotinib	[82]	Phase 3		WBRT + SRS	NS	126	6.1 m
Gefitinib	[23••]	Phase 2	Prior CT		All EGFRact+	41	21.9 m
Gefitinib	[105]	Phase 2	Prior CT or CT + XRT	Palliative care	Chinese NS	40	14 m
Gefitinib	[22]	Phase 2	Prior CT	WBRT	Chinese NS	21	13 m
Gefitinib	[19]	Phase 2	90% prior CT, 44% prior XRT		NS	41	5 m
Gefitinib	[20]	Phase 2	Prior CT	WBRT	NS	59	6.3 m
Icotinib	[37•]	Phase I	25% prior CT	WBRT	All EGFRact+	15	20.8 m
Icotinib	[38]	Phase 2	80% prior CT	WBRT	10/18 EGFRact+	20	22 m

*CT* chemotherapy, *EGFRact+* epidermal growth factor activating mutation, *m* months, *MultiI* multi-institutional, *NS* not screened for EGFRact+, *OS* overall survival, *SRS* radiosurgery, *TKI* tyrosine kinase inhibitor, *WBRT* whole brain radiation therapy, *XRT* radiotherapy

#### First and second generation TKIs

Gefitinib, a first generation TKI, has CSF concentration approximately 1% of serum; however, the concentration may increase with WBRT [17, 18]. The first prospective trial of gefitinib in NSCLC with BrM was conducted in Italy and had a low RR; however, they did find that patients who had previously received WBRT, had better disease control compared to radiation naïve patients [19]. As with all early studies, patients were not molecularly selected for EGFRact+ and gefitinib did not demonstrate a survival benefit [20]. While not molecularly selected, studies conducted in Asian countries enrolling predominately adenocarcinoma had high RR (60% in a Japanese study that had prior radiotherapy and 81% in a Chinese study treated in combination with WBRT) [21, 22]. Subsequent studies in EGFRact+ patients have demonstrated a RR of 87.8% as monotherapy with overall survival (OS) 21.9 months [23••]. Gefitinib is the most common first line TKI in Asian countries and retrospective studies of EGFRact+ enriched populations have supported the use of TKIs as monotherapy without radiation [24, 25•, 26].

Erlotinib is a first generation TKI that is more commonly used in the USA due in part to sustained FDA approval and increased CSF penetration (approximately 5% of serum in patients with BrM which is above the minimum inhibitory concentration) [27–29]. Early prospective trials failed to demonstrate a survival benefit with the addition of erlotinib to radiotherapy in patients with BrM; however, these trials did not screen for EGFRact+ [30–32, 33•]. A subsequent study with a small percentage of patients evaluated for EGFRact+ confirmed CNS response varied by genotype; EGFRwt patients survived 11.8 months; however, OS in EGFRact+ patients was 19.1 months [34]. Current dosing of erlotinib at 150 mg daily is based on inhibition of EGFRwt; however, mathematical modeling suggested pulse dosing strategy could delay development of resistance and CNS progression in EGFRact+ [35]. In 2011, retrospective case series of patients treated with weekly high-dose erlotinib supported a partial CNS response in six of nine patients [36]. Recently, an open-label study of 34 patients treated with a pulse of high-dose erlotinib for 2 days a week and otherwise 50 mg daily [37•]. While this did not prevent the development of T790M resistance, all patients with BrM had intracranial response and no patients with disease progression developed new BrM [37•]. This dosing strategy still needs to be studied in a randomized trial; however, it provides an additional treatment option for those patients with isolated intracranial disease progression. When considering dosing of erlotinib, it is also important to note that smoking decreases the bioavailability of erlotinib [9].

Icotinib, another first-generation TKI, is approved for use in China and has CSF concentration approximately 1% of serum that fluctuates with radiotherapy [38]. Dose escalation studies in combination with WBRT have confirmed safety and RR of 80% in a small phase II of 20 patients (at least 50% with EGFRact+) [39]. There are multiple ongoing trials further examining this agent in combination with radiotherapy for BrM (NCT01926171, NCT02726568, NCT01724801).

Afatinib, a second generation TKI, is unique as an irreversible inhibitor of EGFRact+, EGFRwt, Erb-2, and Erb-4. Likely secondary to its effects on EGFRwt, this agent is associated with greater toxicity; however, dose reduction in the first 6 months due to adverse events was not associated with decreased in progression-free survival (PFS) [40]. Subgroup analysis of LUX-Lung 3 and 6 confirmed CNS activity of this agent and superiority to conventional chemotherapy with increased intracranial PFS if patients had prior WBRT [41]. There is also a case study of five patients who declined WBRT and had complete intracranial response to afatinib [42].

While trials directly comparing first generation TKIs with BrM are ongoing (NCT2714010), retrospective analysis does not support significant survival difference between erlotinib and gefitinib [43–45]. LUX-Lung 7, a prospective comparison of afatinib to gefitinib did support improved time to treatment failure for the use of afatinib over gefitinib; however, there was no difference for 15% patients with asymptomatic BrM [46].

At the time of progression, there is a lack of prospective data to support transitioning between first and second generation TKIs. Current guidelines recommend re-biopsy or cell-free DNA testing at clinically significant disease progression to evaluate for T790M resistance which would guide transitioning to a third-generation TKI. The LUX-Lung 4 study prospectively evaluated transitioning to afatinib in patients progressing on a first-generation TKI and found a response rate of only 8.2%; however, this study excluded patients with active CNS disease [47]. There are reports of intracranial response to erlotinib after progressing on gefitinib [23••, 44, 48] and afatinib [49]. But, there are also retrospective studies of patients with active CNS disease, both of brain metastases and leptomeningeal disease, who progressed on prior treatments with erlotinib or gefitinib but had intracranial response to afatinib [50]. Additionally, there have been reports of patients responding to erlotinib even after development of the T790M [51, 52]. We utilized pulse dosing for isolated CNS progression, as there is evidence that T790M mutations are less common in BrM during pharmacokinetic failure than in systemic disease [51]. At systemic disease progression with identification of T790M resistance mutation, we transition to third-generation TKIs.

#### Third-generation TKIs

In 60% of cases, the development of resistance to a first- or second-generation TKI is due to the acquired point mutation T790M. This mutation is in the ATP binding pocket and prevents inhibition by first- and second-generations TKIs. Osimertinib is a potent irreversible inhibitor of T790M that demonstrated a response rate of over 60% in patients with T790M in early trials leading to the breakthrough designation. Supporting use of osimertinib over conventional chemotherapy, the AURA3 trial demonstrated an impressive PFS of 10.1 months for osimertinib compared to 4.4 months for pemetrexed containing platinum doublet chemotherapy [53••]. This PFS benefit was true even for the over 30% of patients with asymptomatic BrM at enrollment with a hazard ratio of 0.32 [53••]. Intracranial response has also been reported in patients with BrM as well as patients with leptomeningeal disease [54–56]. For patients with T790M, either primary or secondary, we favor the use of osimertinib for the use of EGFRact+ BrM.

#### Future agents

There are multiple TKIs in early clinical phases that are focusing on increased CNS penetration [57•]. One example, the ongoing phase I study of AZD3759, reported preliminary data that 11 of 21 patients had intracranial response [58]. Other ongoing clinical trials of new TKIs in NSCLC with BrM including tesevatinib (NCT02616393) and ASP 8273 (NCT02113813).

### Platinum doublet chemotherapy

Conventional chemotherapy was thought to have a limited role in the treatment of BrM due to concerns about BBB penetration. While not equal to serum, pemetrexed, an antifolate chemotherapy, has CSF concentration only 2% lower than plasma [59]. Systemic RR are similar or lower than intracranial RR in a phase II trials with delayed or concurrent WBRT [60, 61]. Additionally, retrospective analysis found lower rate of symptomatic BrM in NSCLC patients treated with pemetrexed either in first or second line therapy [62]. For patients with advanced adenocarcinoma NSCLC with non-operable asymptomatic BrM, pemetrexed had similar intracranial and extracranial response of 80 and 70%, respectively [63]. While there was limited inclusion of patients with BrM in the large phase III trials, pemetrexed maintenance was shown to improve survival [64]. Based on data from IMPRESS trial, continuing TKI with conventional chemotherapy is not recommended [65]. There are multiple phase III trials to evaluate dose of pemetrexed and use in combination with radiotherapy and TKIs (NCT02284490, NCT02162537, NCT01951469). In patients with non-squamous NSCLC who do not respond to TKIs or at recurrence after osimertinib, platinum doublet chemotherapy of pemetrexed with carboplatin for four to six cycles is recommended for patients with performance status of 0–2. After completion of six cycles, or if side effects prevent further cycles, we utilize pemetrexed as maintenance therapy.

### Immunotherapy

The three immune checkpoint inhibitors approved for use in NSCLC, nivolumab, pembrolizumab, and atezolizumab, have also revolutionized the treatment of NSCLC [66]. Preclinical work supports T cell exhaustion and decreased PDL-1 expression on EGFRact+ tumors [67]. Given the high RR of EGFRact+ tumors to TKIs, clinical trials of checkpoint inhibitors have excluded EGFRact+ patients, as well as patients with active CNS disease. Given these limitations, the use of immunotherapy in EGFRact+ NSCLC with BrM is limited to case series and small trials that have demonstrated efficacy [68••], although also with significant neurologic side effects [69]. There are ongoing trials combining immunotherapy with TKIs in EGFRact+ NSCLC (NCT02085070). At this time, checkpoint inhibitors are reserved for use after TKIs and usually not until progression on conventional chemotherapy unless there is high expression of PDL-1.

### Antiangiogenic therapy

Bevacizumab (BEV) has demonstrated a survival benefit in combination with platinum doublet chemotherapy in non-squamous NSCLC, although these trials excluded patients with known CNS metastasis given concerns for intracranial hemorrhage [70]. Subsequently, the safety of the addition of BEV in patients with non-squamous NSCLC BrM, even in the setting of full anticoagulation, has been demonstrated [71•]. For this reason, addition of BEV to platinum doublet chemotherapy is recommended in non-squamous NSCLC. While it excluded patients with BrM, an open-label phase II study comparing the addition of BEV to erlotinib in first line therapy demonstrated a PFS benefit compared to erlotinib alone; survival data has not matured and a phase III study is ongoing [72]. At this time, the use of BEV in treatment of BrM is primarily in the management of steroid refractory vasogenic edema and for the management of radiation necrosis with recognition that there may be a benefit to tumor control as well.

## Local therapy

### Surgery

The survival and QOL benefits of surgery for treatment of a single symptomatic BrM are based on randomized trials from the 1990s with a population of primarily lung cancer patients. These trials were randomized to surgical resection and WBRT or WBRT alone and found not only a survival benefit to the surgical group, but also benefit in functionally independent survival [73, 74]. While a more recent retrospective study of patients with BrM at initial diagnosis of NSCLC did not demonstrate a survival benefit to surgery, surgery did improve QOL when presenting with neurologic symptoms [75]. Advances in neurosurgical techniques allow access to more lesions with low surgical complications and morbidity of only 1.8% at high volume centers [76]. Given the response of BrM to radiotherapy and systemic therapies, we reserve surgery for large (greater than 3 cm), symptomatic, accessible BrM or if additional histologic diagnosis is required.

### Radiotherapy

Radiotherapy has been the foundation of treatment of solid tumor BrM for decades; however, the type and timing of radiotherapy remains highly variable in clinical practice. Given EGFRact+ is a positive prognostic factor with high RR to TKIs, we typically reserve the use of radiotherapy until after TKI response can be evaluated. The clinical evidence to support this decision is limited to multiple small studies of TKIs or conventional chemotherapy as monotherapy (Table 2). These studies have supported the use of systemic therapies to delay radiation therapy from 12.6 to 17.9 months [25•, 48, 77••]. A recently reported retrospective study suggests increased survival with combination of up-front SRS and TKI compared to salvage strategy [78••]. Review of this literature provides a foundation for our practice of delaying WBRT to evaluate for response to systemic agents.

**Table 2:** Clinical trials evaluating the use of TKIs without radiotherapy in EGFRact+ NSCLC

TKI studied		Type of study	Additional treatment	*N*	EGFR status	ORR	iRR	OS
Erlotinib	[105]	Phase 2	Prior CT	48	8/23 EGFRact+	58.3%	NR	18.9 m
Erlotinib	[78••]	Retrospective	1) SRS + TKI 2) TKI monotherapy	50	All EGFRact+	1)88% 2)76%	NR	1) 58.4 2)19.4
Gefitinib	[105]	Phase 2	1) PriorCT+XRT 2) Prior CT	40	Chinese NS	1)36% 2)31%	38%	15 m
Gefitinib	[23••]	Phase 2	Prior CT	41	All EGFRact+	87.8%	65.80%	21.9 m
Erlotinib	[23••]	Phase 2	At progression on gefitinib	12	All EGFRact+	58.3%	NR	NR
Gefitinib	[106]	Retrospective	1) Concurrent WBRT 2) TKI monotherapy	90	12/20 EGFRact+	1) 71.4% 2)60%	1) 71% 2)40%	1) 23.4 2)14.8
Gefitinib or erlotinib	[25•]	Retrospective	Prior CT	43	All EGFRact+	72%	57%	23.6 m
Gefitinib or erlotinib	[26]	Retrospective	1) Prior XRT 2) TKI monotherapy	121	All EGFRact+	1)83.1% 2) 85.5%	1)79.7% 2)59.7%	NM but iPFS 1) 16.6 m 2) 21 m
Gefitinib or erlotinib	[45]	Phase 2	Prior CT	28	All EGFRact+	83%	83%	15.9 m
Gefitinib or erlotinib	[95]	Retrospective	1) TKI + XRT 2) TKI monotherapy	89	All EGFRact+	NR	NR	1) 26 m 2) 21.5 m
Gefitinib or erlotinib	[107]	Retrospective	1) WBRT + TKI 2) TKI monotherapy	81	All EGFRact+	NR	NR	1) 18.5 m 2) 17 m
Gefitinib or erlotinib	[96]	Retrospective	1) SRS+ TKI 2) TKI monotherapy and salvage XRT	162	All EGFRact+	NR	NR	1) 40.8 m 2) 20.5 m

*CT* chemotherapy, *EGFRact*+ epidermal growth factor activating mutation, *m* months, *iPFS* intracranial progression free survival, *iRR* intracranial response rate, *NM* data not matured, *NS* not screened for EGFRact+, *NR* not reported, *PFS* progression free survival, *ORR* overall response rate, *OS* overall survival, *RR* response rate, *TKI* tyrosine kinase inhibitor, *XRT* radiotherapy

#### Whole brain radiation therapy

Due to limited CNS penetration of older systemic therapies, WBRT, with or without surgery, was the cornerstone of management of BrM, based on evidence that patients were less likely to die of neurologic causes despite a lack of survival benefit [79]. Multiple studies evaluating the safety of chemotherapy in conjunction with WBRT demonstrated improved CNS control; however, there was no improvement in OS. No survival benefit has been demonstrated when comparing early WBRT to delayed following conventional chemotherapy [80]. Small studies examining the use of TKIs and WBRT have provided conflicting results and it remains unclear if addition of TKI to radiotherapy improved survival [81] or could possibly have deleterious effects [82]. In contrast, clinical trials have reported that patients were limited in receiving subsequent systemic treatment due to deteriorations in performance status after WBRT [26]. Meta-analysis also confirms more side effects with radiotherapy compared to TKIs alone [83•].

The benefits of radiation therapy must be weighed with not only the short-term side effects, but given survival of years for many patients with EGFRact+ NSCLC, the subacute and long-term cognitive effects of radiation [12]. For patients with poor functional status, WBRT has no significant survival or QOL benefit compared to best supportive care [84••]. Applications of these studies to the EGFRact+ NSCLC population are limited and there are multiple ongoing studies evaluating the use of WBRT in combination with TKIs [85]. We reserve WBRT for patients who have intracranial progression refractory to all systemic therapies that are not candidates for surgery or SRS, if performance status permits.

#### Stereotactic radiosurgery

SRS provides high-dose radiation to the target while minimizing exposure to surrounding normal brain tissue. The number of BrM that can be treated with SRS is expanding with a recent trial confirming non-inferiority with SRS for up to 10 metastases compared to WBRT [86]. The use of SRS is limited based on location and size of lesions as well as degree of vasogenic edema, so that it is primarily offered to patients with only a few, small BrM (<3 cm given decline in local control) [87].

Initially, clinical trials evaluated the addition of SRS to WBRT and found improved survival only for patients with a single BrM, but improved performance status of all patients [88]. Subsequent retrospective studies had mixed findings with some demonstrating improved CNS control however no improvement in OS and others actually finding that SRS following WBRT decreased survival [89, 90]. Recent post hoc analysis of the EORTC 22952 trial failed to find a benefit to adjuvant WBRT [91]. Additionally, combination SRS and WBRT has a significant impact on learning and memory at 4 months compared to SRS alone [92]. For these reasons, clinical practice has moved towards the use of SRS alone.

There are multiple hypotheses suggesting that radiation can increase the efficacy of TKIs possibly by improving BBB penetration or radiosensitizing the tumors with TKIs [93, 94]. There was a small phase III study that did not find a survival benefit to up-front SRS compared to conventional chemotherapy; however, this trial was closed early due to the approval of TKIs [77••]. Phase II and retrospective studies have supported the use of TKIs as monotherapy to delay radiation therapy, although these studies were primarily with WBRT or mixture of radiotherapy (Table 2). Retrospective studies of the use of up-front SRS with TKI support a survival benefit over the use of TKI monotherapy with salvage radiotherapy [78••, 95, 96]. A devastating long-term effect of radiation, leukoencephalopathy, radiographically presents as diffuse periventricular white matter changes [97]. The rate of leukoencephalopathy following SRS is lower than WBRT at all time points but is up to 84% at 4 years [98••]. Recognizing the risk of radiation, at this time, we favor the use of SRS in combination with TKIs for BrM <3 cm given appropriate performance status.

### Management of subacute and late neurologic complications of radiotherapy

Radiation necrosis clinically presents with general and neurologic decline due to brain edema surrounding a growing necrotic core. In other brain tumor populations, radiation necrosis has been associated with similar survival as recurrent disease. Steroids are used as first line management of radiation necrosis. However, if there is no significant improvement with steroids, we transition to management with BEV utilizing a standard 6-week protocol [99].

Cognitive impairment can be seen as a result of radiation therapy with variable reversibility. Acute cognitive complaints within 1 month of radiation are most commonly related to fatigue or vasogenic cerebral edema and can be managed conservatively. Cognitive slowing, executive dysfunction, and short-term memory deficits seen in the 1 to 6 months following radiotherapy may resolve with time and lifestyle modification. There is limited data that memantine, an NMDA partial antagonist, hyperbaric oxygen and hippocampal sparing WBRT may prevent this impairment [100–102].

Late cognitive impairment, leukoencephalopathy, increases as a function of time following both SRS and WBRT [98]. These changes are irreversible and associated with both demyelinating and cerebrovascular pathology. For these reasons, it is more common in patients over the age of 70 with vascular disease or collagen disorders. This has been seen more commonly in WBRT and clinically is a driving force for delaying WBRT.

Hypopituitarism develops in virtually all patients with radiotherapy near the pituitary and 20% within 5 years. In long-term survivors, monitoring for pituitary function after radiation therapy is also important.

### Emerging therapies

There are multiple ongoing trials as described above to clarify the use of currently available agents. In addition, for further development of TKIs, the National Lung Matrix Trial is using biomarkers to guide targeted drug therapies. The use of irinotecan, etoposide, and ATR kinase inhibitors is also under investigation. When patients progress on TKI and conventional chemotherapy, we encourage offering additional clinical trials to the patients.
